# Supplementary material for: Nitrogen and phosphorus trends in lake sediments of China may diverge
Source: Nat Commun. 2024 Mar 26;15:2644. doi: 10.1038/s41467-024-46968-4 (PMC10966067; doi:10.1038/s41467-024-46968-4)
Supplement: Supplementary file 1 — Supplementary Information [file 41467_2024_46968_MOESM1_ESM.pdf]

*Supplementary Information for*

# Nitrogen and phosphorus trends in lake sediments of China may diverge

Ji Panpan[1], Chen Jianhui[1*], Chen Ruijin[1], Liu Jianbao[2], Yu Chaoqing[3], Chen Fahu[1,2,4]

[1]MOE Key Laboratory of Western China's Environmental System, College of Earth and Environmental Sciences, Lanzhou University, Lanzhou 730000, China

[2]ALPHA, State Key Laboratory of Tibetan Plateau Earth System, Environment and Resources (TPESER), Institute of Tibetan Plateau Research (ITPCAS), Chinese Academy of Sciences (CAS), Beijing 100101, China

[3]College of Ecology and Environment, Hainan University, Haikou, Hainan 570228, China

[4]College of Resources and Environment, University of Chinese Academy of Sciences, Beijing 100049, China

**Supplementary Information file includes:**
**Supplementary figures 1-13**
**Supplementary tables 1-4**

Supplementary Fig. 1 | Age-depth models of 8 lake sediment cores from the Inner Mongolian Plateau and Xinjiang. ....4
Supplementary Fig. 2 | Temporal distribution of turning points of the TN and TP records of the 69 lakes in China. ....5
Supplementary Fig. 3 | Social and economic development in China. ....6
Supplementary Fig. 4 | Mean variance plots of the variation ratio of TN, TP, and ΔTN, ΔTP for the 69 lakes from the six districts of China, in relation to geographical location. ....7
Supplementary Fig. 5 | Spatial distribution of agricultural productivity, population density, and GDP for China. ....7
Supplementary Fig. 6 | Climate records for the six districts of China and averaged total nitrogen (TN) and total phosphorus (TP) trends for 1850–2014. ....8
Supplementary Fig. 7 | Cause of the difference in lake nutrient levels between the Yunnan-Guizhou Plateau and the Eastern Plain. ....9
Supplementary Fig. 8 | Relationship between nutrient accumulation and lake volume for the studied lakes. ....10
Supplementary Fig. 9 | Maps showing differences in the proximity of the studied lakes to cultivated land (a), and N dry deposition rate (b), N wet deposition rate (c) of China. ....10
Supplementary Fig. 10 | Past and predicted future nitrogen (N) and phosphorus (P) fertilizer consumption in the lake districts of China. ....11
Supplementary Fig. 11 | Past and predicted future N deposition in the lake districts of China (a) under different social development scenarios (b-d). ....11
Supplementary Fig. 12 | Comparison of AIC and BIC in different models and combination of model variables (a), factors information involved in model construction (b), overfitting and model distortion with the addition of new factors (c). ....12
Supplementary Fig. 13 | Model prediction results of historical changes in sedimentary N, P concentrations in each of the six lake districts of China (a-f). ....13
Supplementary Tab. 1 | Details of the 69 lake records from China used in this study. ....14
Supplementary Tab. 2 | List of the datasets used in this study. ....16
Supplementary Tab. 3 | Correlations between factors involved in model construction and other factors. ....17
Supplementary Tab. 4 | Model fitting results for the six districts of China. ....19
Supplementary References ....20

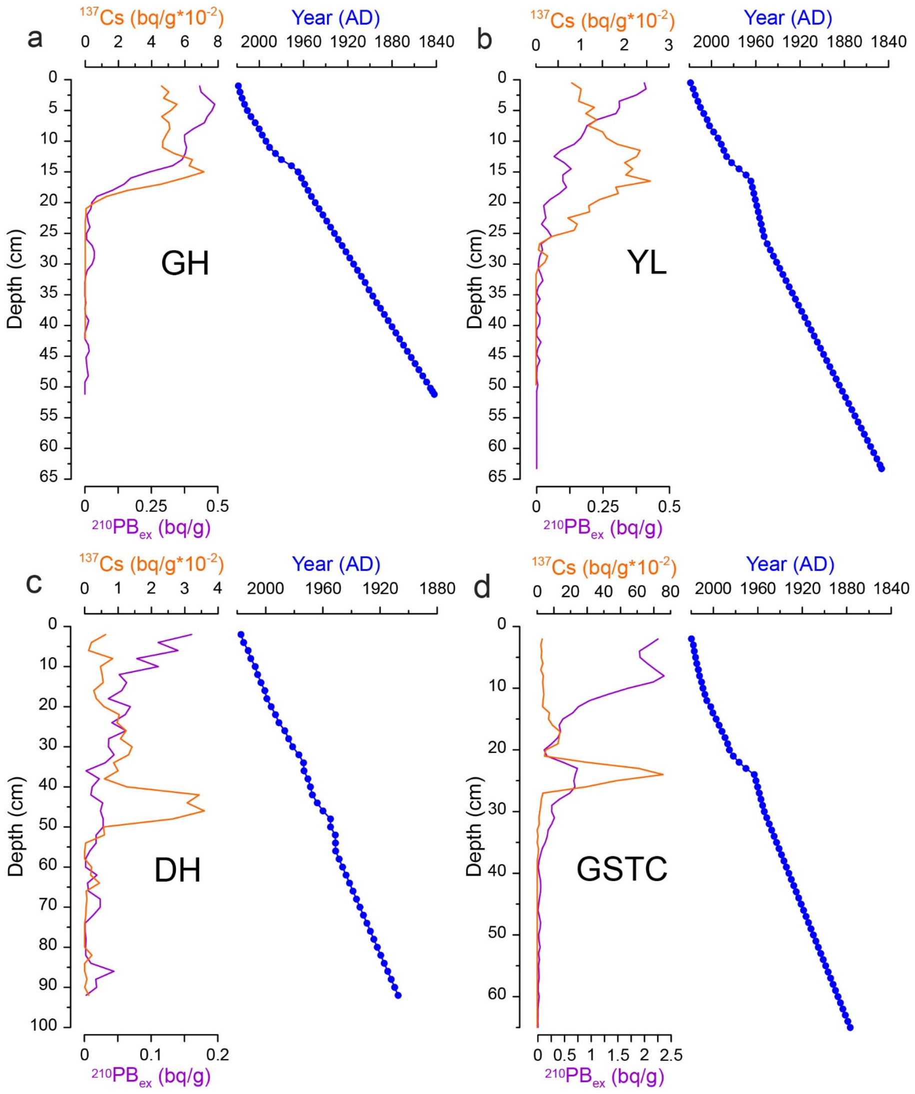
a
b
c
d
GH
YL
DH
GSTC
137Cs (bq/g*10-2)
210PBex (bq/g)
Year (AD)
Depth (cm)

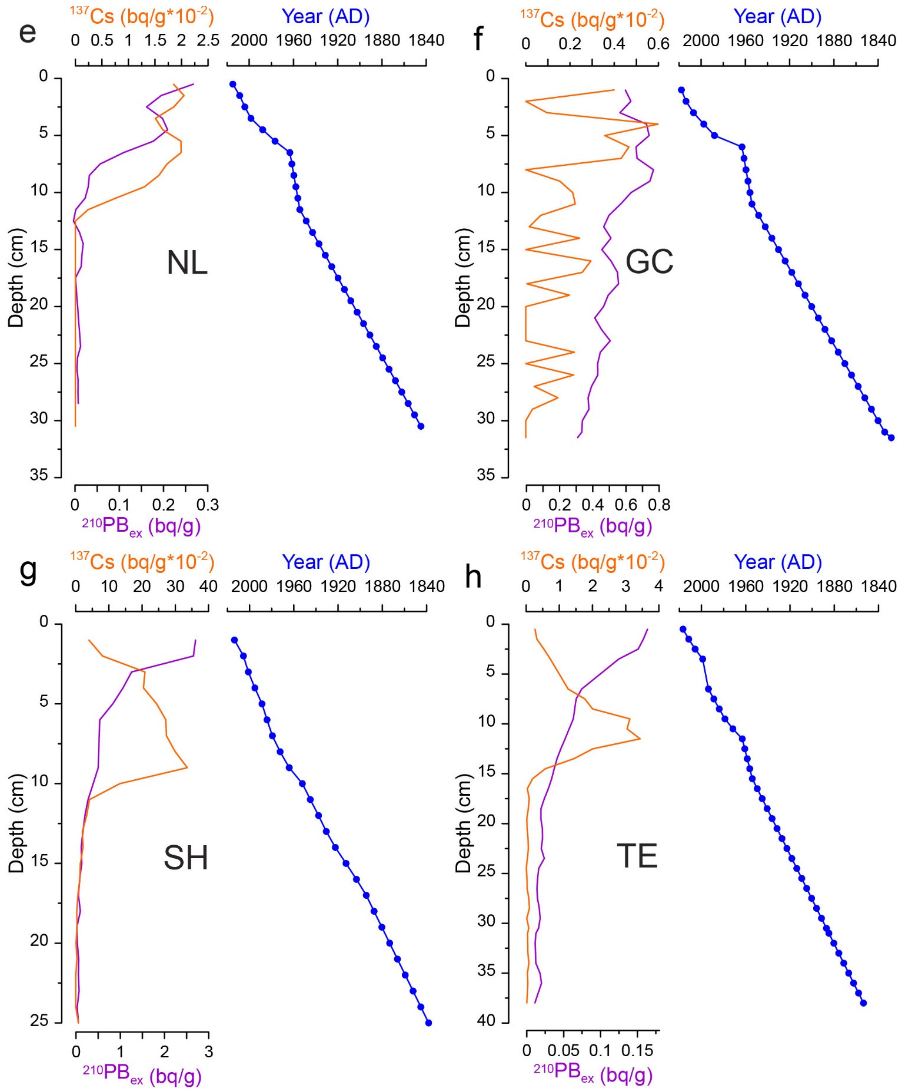


**Supplementary Fig. 1 | Age-depth models of 8 lake sediment cores from the Inner Mongolian Plateau and Xinjiang.** 8 lake cores include: (a) Gonghai lake (GH)[1], (b) Yueliang Lake (YL)[1], (c) Daihai lake (DH), (d) Guanshantianchi lake (GSTC), (e) Nalin Lake (NL), (f) Gouchi lake (GC), (g) Shuanghu lake (SH) and (h) Tianehu lake (TE). $^{210}$Pb and $^{137}$Cs assay was used for dating[2]. The peak in $^{137}$Cs was taken as 1963 yr AD, the ages were computed by using the $^{210}$Pb constant rate of supply (CRS) model span the range of 1840–2020. And the basal age was calculated using the average sediment accumulation rate of the model results for the upper part of the cores. Source data are provided as a Source Data file.

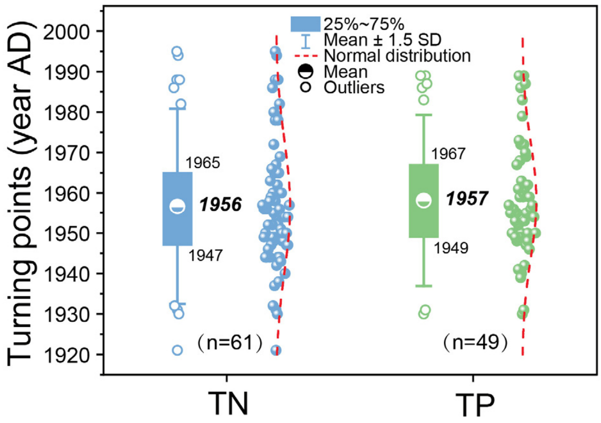


**Supplementary Fig. 2 | Temporal distribution of turning points of the TN and TP records of the 69 lakes in China.** The results are based on 61 TN records (1 record undetected) and 49 TP records. The shift time nodes for TN and TP are compatible with a normal distribution (red broken lines). The mean turning points for TN is 1956 and that for TP is 1957. The error bars are the mean ± 1.5 SD. Source data are provided as a Source Data file.

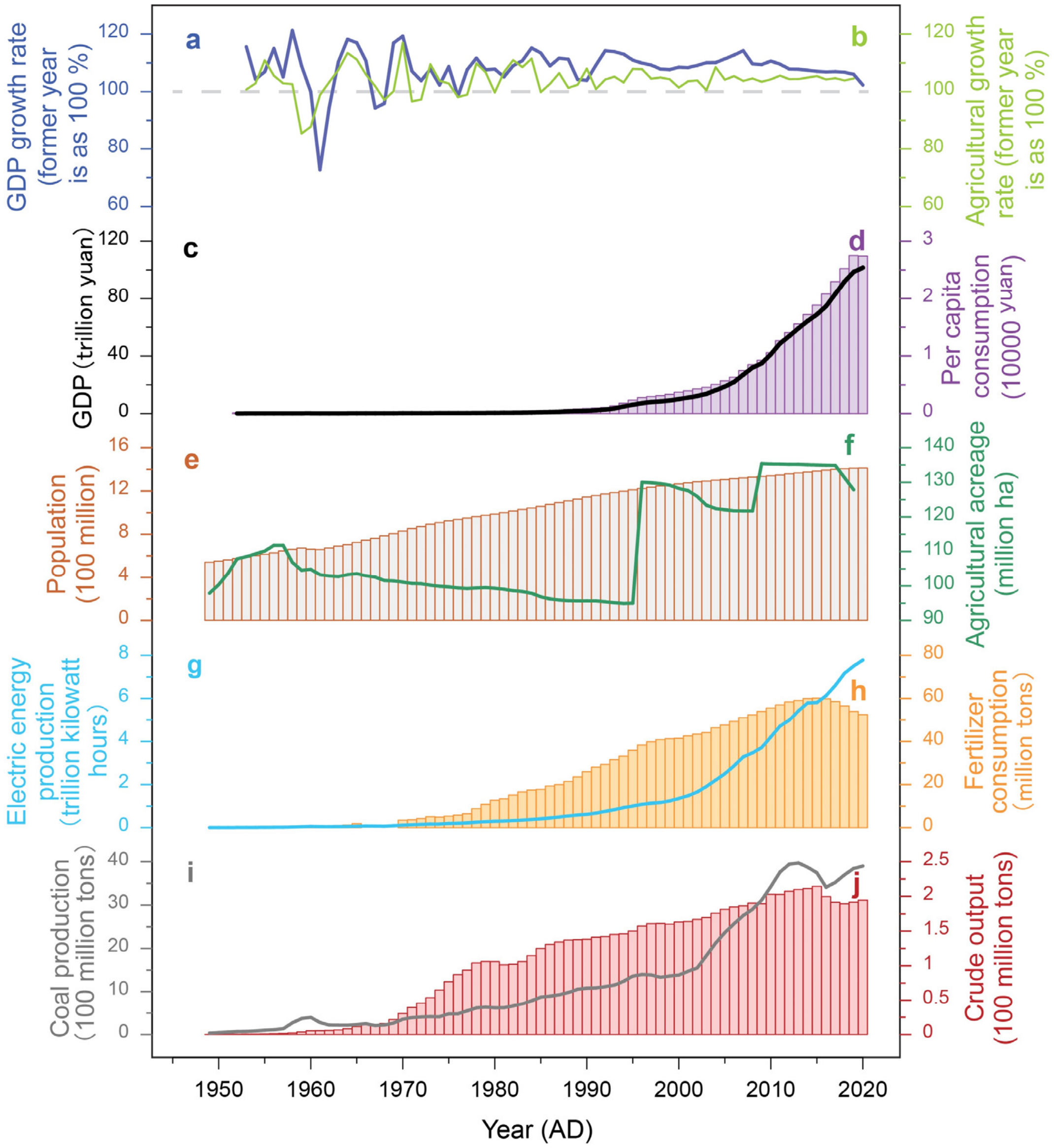


**Supplementary Fig. 3 | Social and economic development in China.** (a) GDP growth rate (the value of the previous year is taken as the baseline), (b) Agricultural growth rate (the value of the previous one year is taken as the baseline), (c) GDP, (d) Per capita consumption, (e) Human population, (f) Electricity production, (g) Agricultural acreage, (h) Fertilizer consumption, (i) Coal production, (j) Crude oil output. (All data are from the National data of China: stats.gov.cn). Source data are provided as a Source Data file.

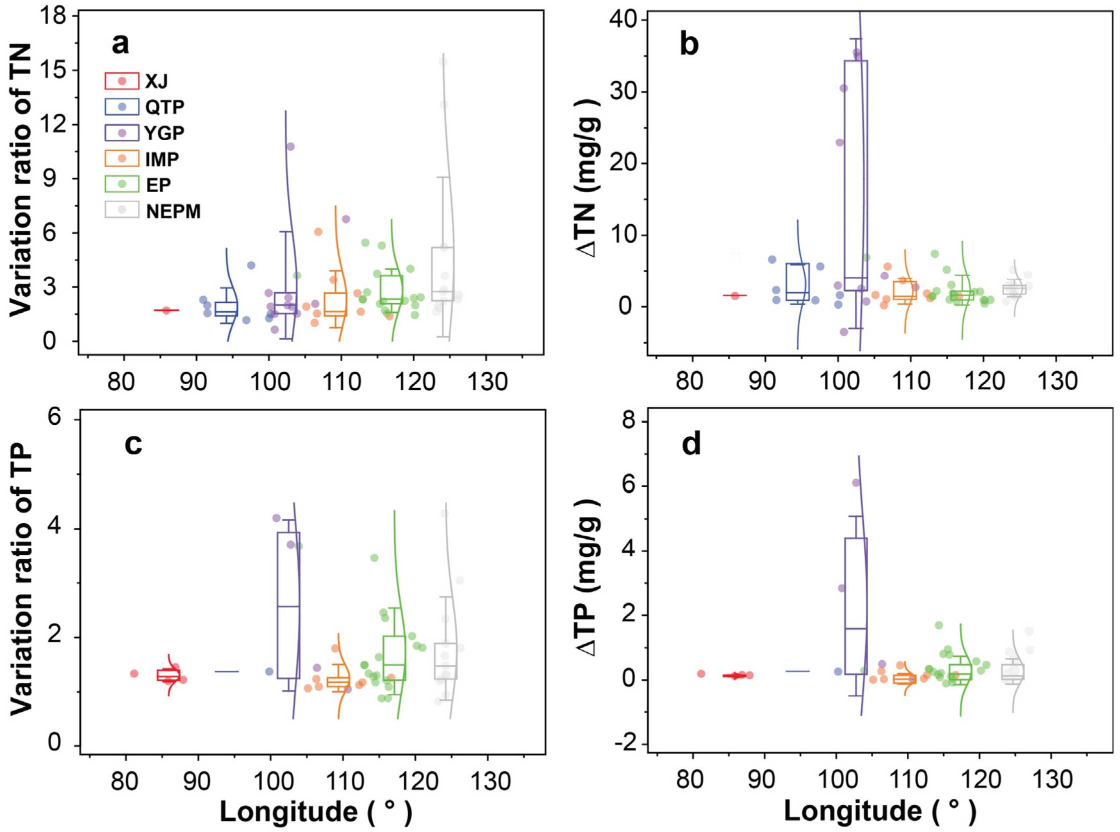


**Supplementary Fig. 4 | Mean variance plots of the variation ratio of TN, TP, and ΔTN, ΔTP for the 69 lakes from the six districts of China, in relation to geographical location.** (a, c) Variation ratio of TN, TP. (b, d) ΔTN, ΔTP. QTP: Qinghai-Tibetan Plateau (I), XJ: Xinjiang (II), IMP: Inner-Mongolia Plateau (III), NEPM: Northeast Plain and Mountains (IV), EP: Eastern plain (V), YGP: Yunnan-Guizhou Plateau (VI). The horizontal line in the box is the median line, the error bars are the 1.5 IQR. Source data are provided as a Source Data file.

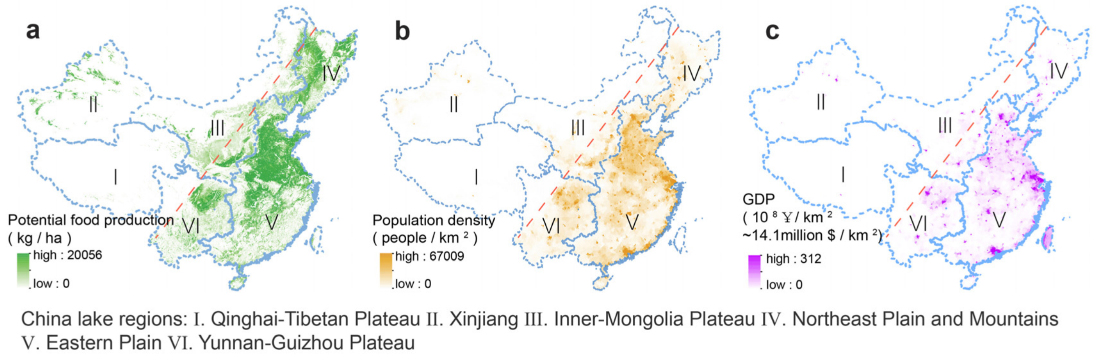


**Supplementary Fig. 5 | Spatial distribution of agricultural productivity, population density, and GDP for China.** (a) Potential food production in 2010, (b) Population density in 2019, (c) GDP in 2019. Note the relationship between these indicators and the Hu Huanyong line (red dashed line)[3], reflecting the natural geographical environment. All data and map data are available from the Resource and Environment Science and Data Center (a Potential food production: https://www.resdc.cn/DOI/DOI.aspx?DOIID=43, b Population density: https://www.resdc.cn/DOI/DOI.aspx?DOIID=32, c GDP: https://www.resdc.cn/DOI/DOI.aspx?DOIID=33).

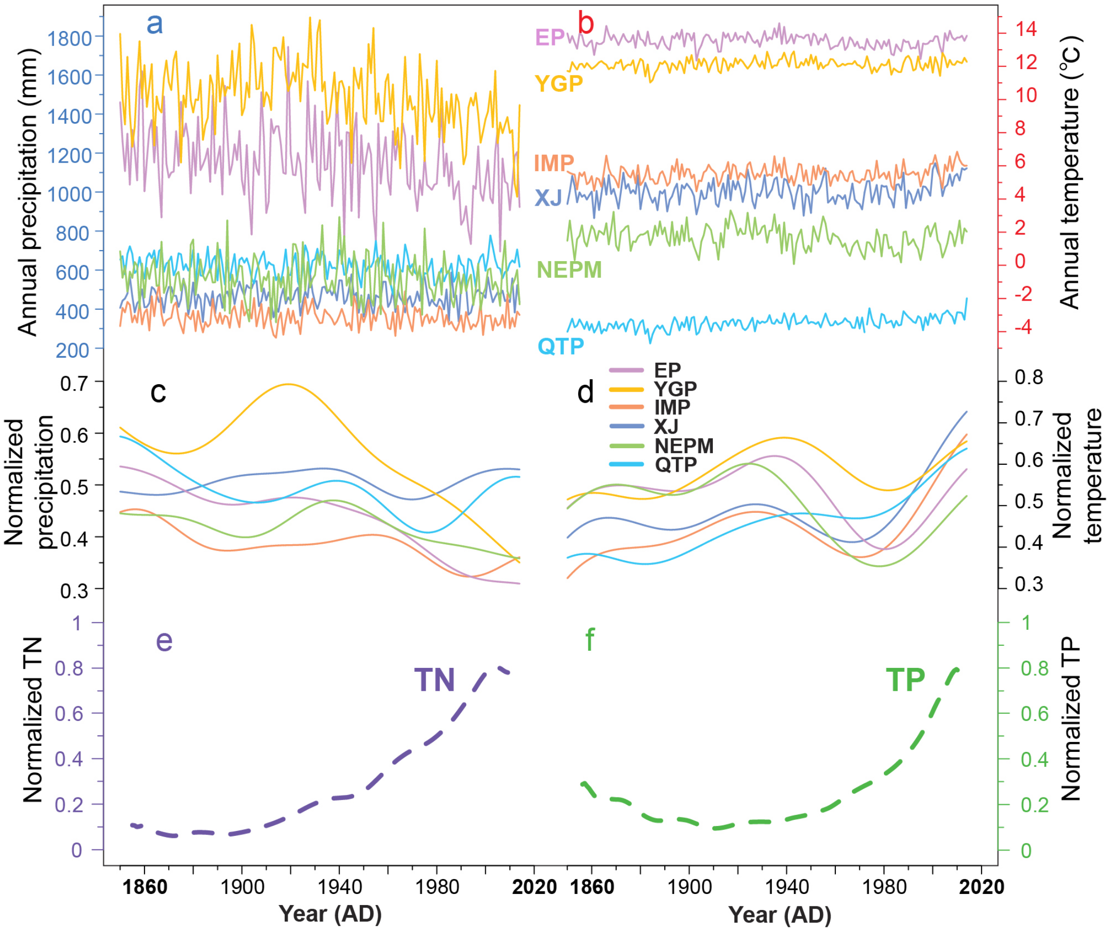


**Supplementary Fig. 6 | Climate records for the six districts of China and averaged total nitrogen (TN) and total phosphorus (TP) trends for 1850–2014.** Annual precipitation (a) and temperature (b), smoothed and normalized records of precipitation (c) and temperature (d) for the six districts of China. Averaged records of TN (e) and TP (f). The smoothed records are normalized to 0–1 and smoothed with a 20-year window. QTP: Qinghai-Tibetan Plateau (I), XJ: Xinjiang (II), IMP: Inner-Mongolia Plateau (III), NEPM: Northeast Plain and Mountains (IV), EP: Eastern plain (V), YGP: Yunnan-Guizhou Plateau (VI). Source data are provided as a Source Data file.

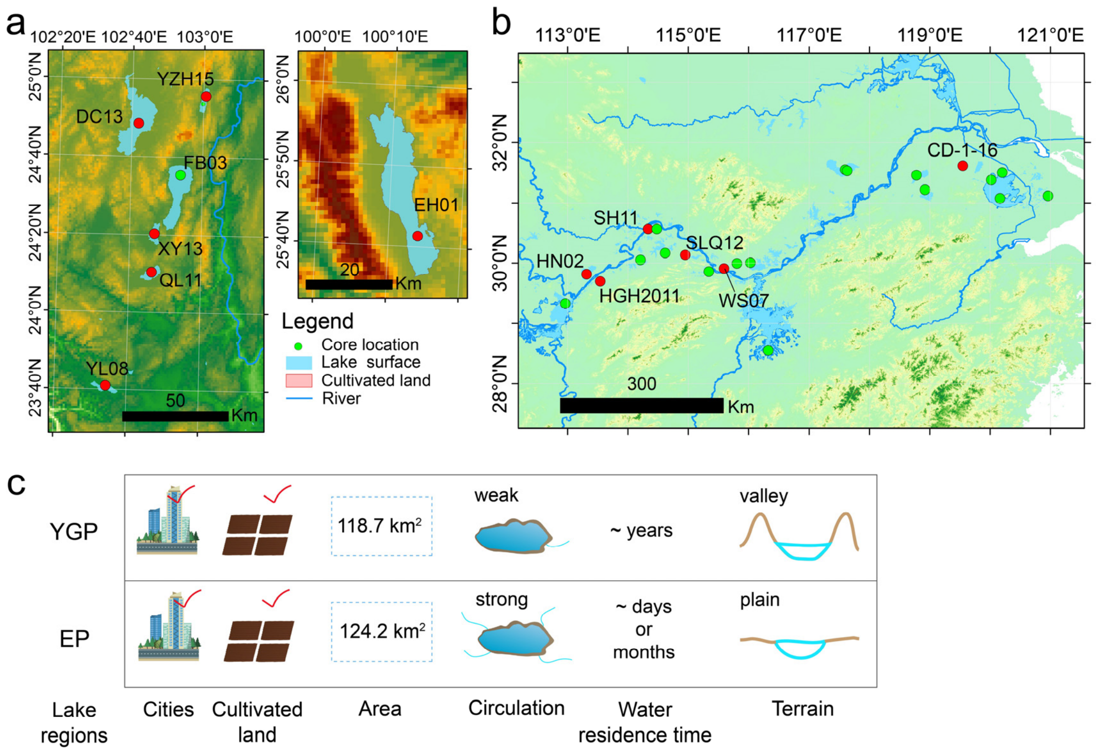


**Supplementary Fig. 7 | Cause of the difference in lake nutrient levels between the Yunnan-Guizhou Plateau and the Eastern Plain.** (a) Locations of lakes and sediment cores on the Yunnan-Guizhou Plateau (YGP, VI). Lakes with high TN concentrations are Dianchi (DC13), Qiluhu (QL11), Yilonghu (YL08), Yangzonghai (YZH15), and Erhai (EH01), red points in (a). After 2000, their TN concentrations exceeded 40 mg/g. The highest TP concentration is in Xingyun Lake (up to 8 mg/g). (b). Locations of lakes and sediment cores in the Eastern Plain (EP, V). Lakes with higher nutrient concentrations are: Honghu (HN02), Huanggai (HGH2011), Shahu (SH11), Sanliqi (SLQ12), Wushan (WS07), and Changdang (CD-1-16), red points in (b). (c) Comparison of potential factors influencing lake nutrient levels between YGP and EP. Both districts have similar climatic condition (relatively high annual temperature and precipitation), but nutrient accumulation in the EP lakes is much lower than in the YGP lakes. All the studied lakes in the YGP and EP are located within densely populated areas, but the EP lakes have superior connectivity with the surrounding drainage system and strong water circulation. Hence, the YGP lakes have long residence times which promoted nutrient accumulation. The digital elevation model used in (a) and (b) is available from the Resource and Environment Science and Data Center (https://www.resdc.cn/data.aspx?DATAID=284). The figure (c) has been designed using resources from Freepik (Cities: https://www.freepik.com/free-vector/urban-buildings-cityscape-view-scenarios_4794195.htm) and Integration and Application Network (ian.umces.edu/media-library) (Cultivated land: originally published by Tracey Saxby. Field: ploughed (https://ian.umces.edu/media-library/field-ploughed/). Released under a Creative Commons Attribution-ShareAlike 4.0 International (CC BY-AS 4.0). Lakes: originally published by Tracey Saxby. Pond 2 (https://ian.umces.edu/media-library/pond-2/). Released under a Creative Commons Attribution-ShareAlike 4.0 International (CC BY-AS 4.0)). The elements have been reorganized and modified.

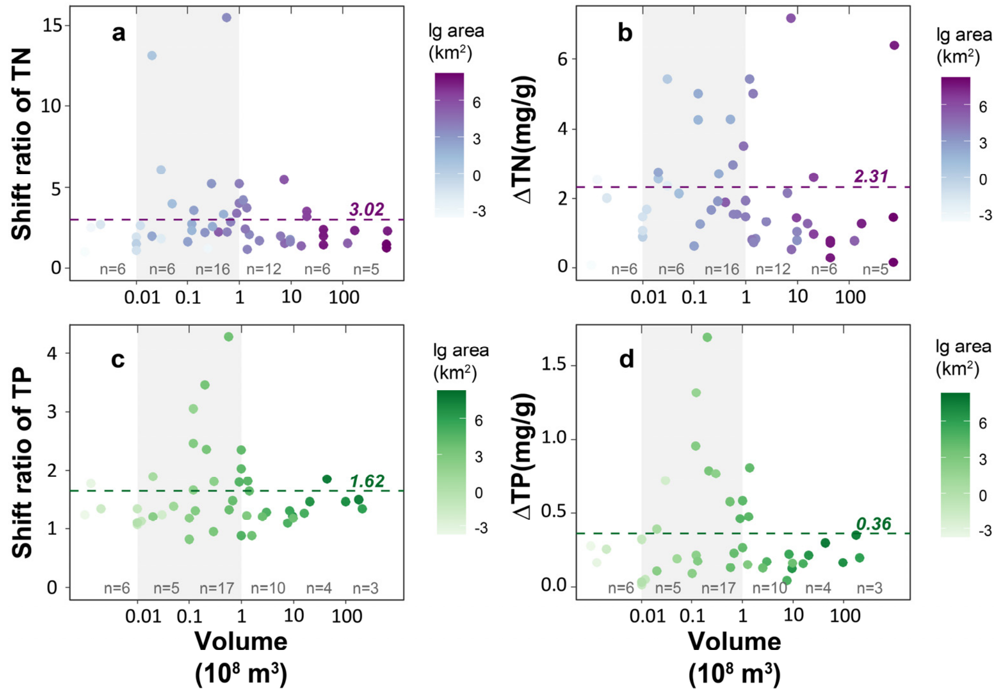


**Supplementary Fig. 8 | Relationship between nutrient accumulation and lake volume for the studied lakes.** (a, c) Shift ratio of TN, TP versus lake volume. (b, d) ΔTN, ΔTP versus lake volume. Horizontal broken lines indicate the average. Except for the lakes of the Yunnan-Guizhou Plateau, lakes with above-average values tend to have volumes in the range of 0.01–1 ×$10^8$ $m^3$ (indicated by the grey shading). Source data are provided as a Source Data file.

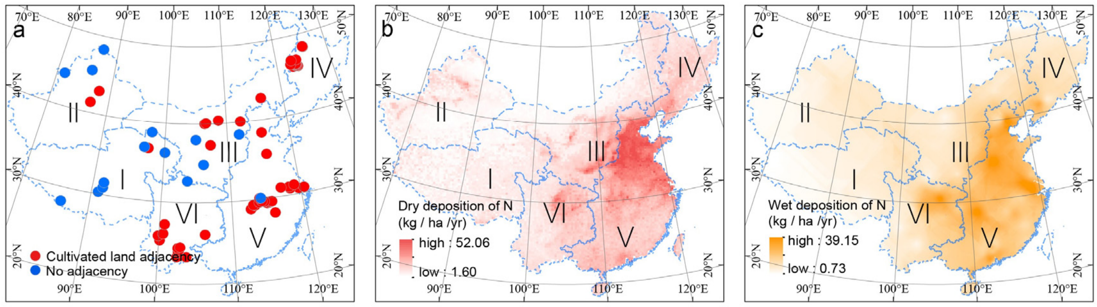


China lake regions: I. Qinghai-Tibetan Plateau II. Xinjiang III. Inner-Mongolia Plateau IV. Northeast Plain and Mountains V. Eastern Plain VI. Yunnan-Guizhou Plateau

**Supplementary Fig. 9 | Maps showing differences in the proximity of the studied lakes to cultivated land (a), and N dry deposition rate (b), N wet deposition rate (c) of China.** In (a) 54 lakes (red dots) out of the total of 69 lakes have adjacent cultivated land. In (b) and (c), the Eastern Plain and Yunnan-Guizhou Plateau have a higher intensity of wet and dry N deposition[4]. The base map is the standard map of China: GS(2019)1823.

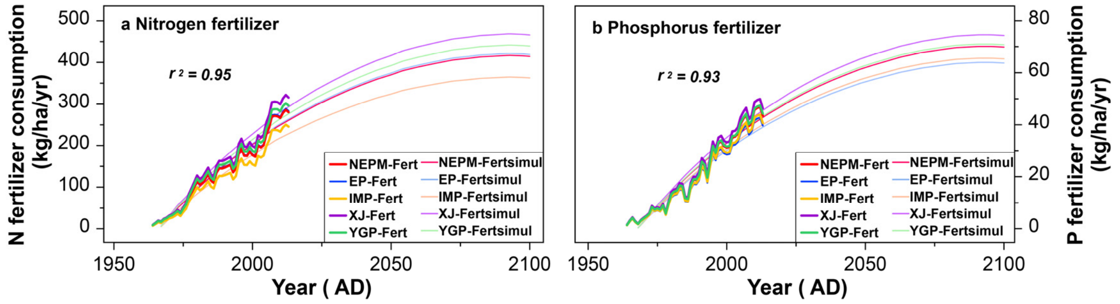


**Supplementary Fig. 10 | Past and predicted future nitrogen (N) and phosphorus (P) fertilizer consumption in the lake districts of China.** The estimates (-Fertsimul) of (a) nitrogen fertilizer consumption and (b) phosphorus fertilizer consumption are based on historical fertilizer consumption (-Fert)[5], and future trends of the projections of global fertilizer consumption[6]. Although the results of future trends are only suitable for relatively crude model construction and calculations[6]. Fertilizer consumption data for the Qinghai-Tibetan Plateau are not available in global datasets[6], and the Qinghai-Tibetan Plateau data were obtained from the National Bureau of Statistics of China. QTP: Qinghai-Tibetan Plateau (I), XJ: Xinjiang (II), IMP: Inner-Mongolia Plateau (III), NEPM: Northeast Plain and Mountains (IV), EP: Eastern plain (V), YGP: Yunnan-Guizhou Plateau (VI). Source data are provided as a Source Data file.

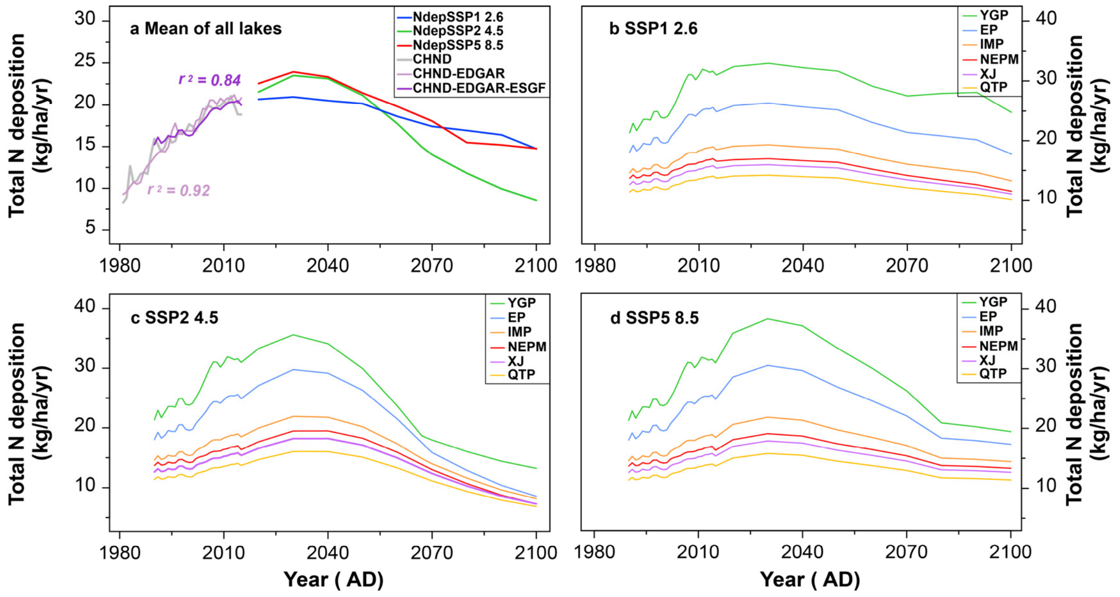


**Supplementary Fig. 11 | Past and predicted future N deposition in the lake districts of China (a) under different social development scenarios (b-d).** The N deposition estimates for China are used as an historical reference (CHND)[4]. The N deposition model (CHND-EDGAR-ESGF) was built using EDGAR[7] and the input4MIPS ESGF N emission datasets[8], based on CHND (see Methods for details). The model (CHND-EDGAR-ESGF, dark purple line) was used to estimate the trends of N deposition for each district, and to simulate future N accumulation trends for the lakes. QTP: Qinghai-Tibetan Plateau (I), XJ: Xinjiang (II), IMP: Inner-Mongolia Plateau (III), NEPM: Northeast Plain and Mountains (IV), EP: Eastern plain (V), YGP: Yunnan-Guizhou Plateau (VI). Source data are provided as a Source Data file.

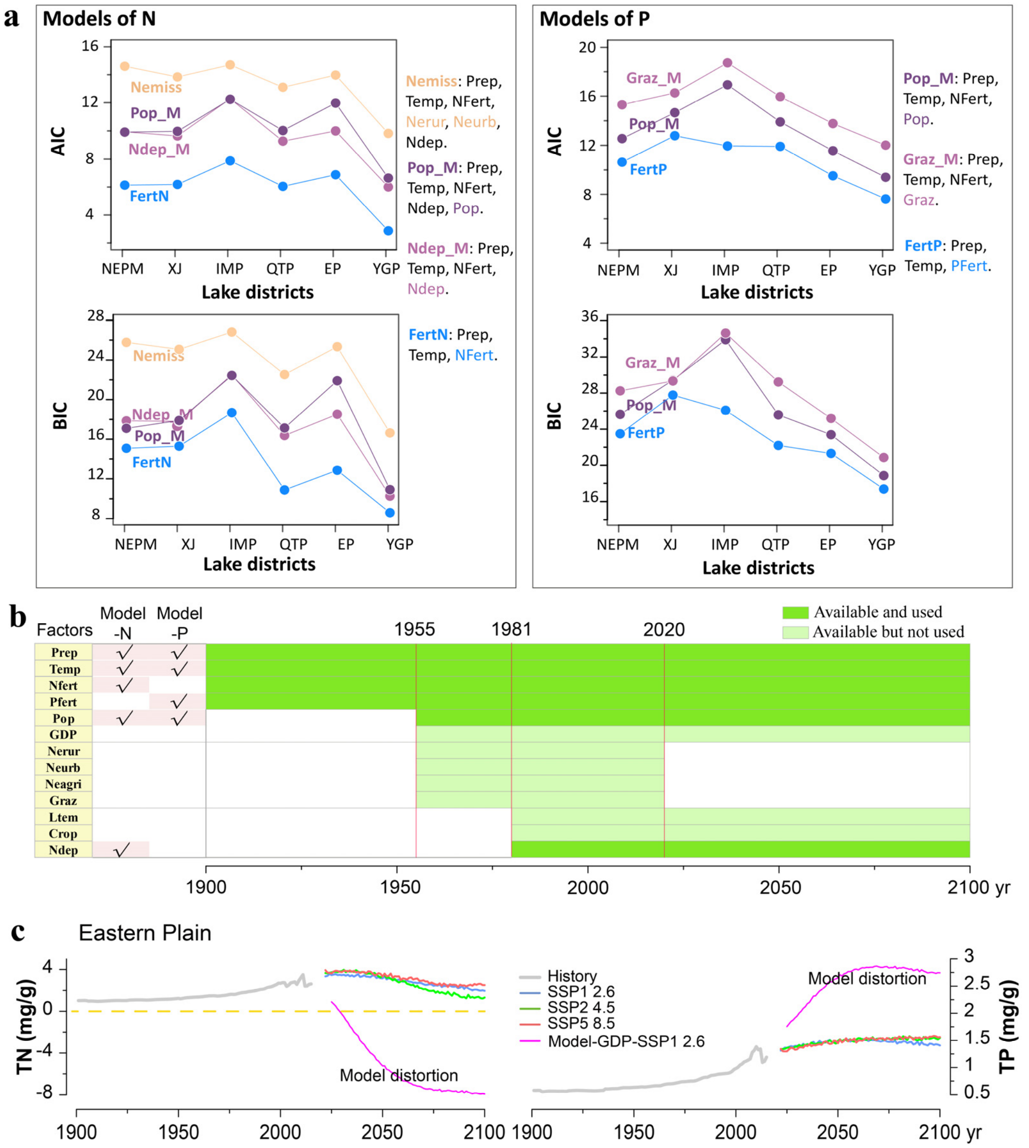


**Supplementary Fig. 12 | Comparison of AIC and BIC in different models and combination of model variables (a), factors information involved in model construction (b), and overfitting, model distortion with the addition of new factors (c).** The variables include: Prep: precipitation, Temp: temperature, Nfert: fertilizer consumption of N, Pfert: fertilizer consumption of P, Pop: population, GDP: Gross Domestic Product, Nerur: N emission from rural, Neurb: N emission from urban, Neagri: N emission from agriculture, Graz: amount of livestock farming, Ltem: lake temperature, Crop: percent of cropland in lake basin, Ndep: N deposition. (a), left panel, N models include: FertN (Prep, Temp, Nfert), Ndep_M (Prep, Temp, Nfert, Ndep), Pop_M (Prep, Temp, Nfert, Ndep, Pop), and Nemiss (Prep, Temp, Nfert, Nerur, Neurb, Ndep), four models, respectively, considering the relative independence of the factors used in each model. (a), right panel, P models include: FertP (Prep, Temp, Pfert), Pop_M (Prep, Temp, Pfert, Pop), and Graz_M (Prep, Temp, Pfert, Pop, Graz), three models, respectively, considering the relative independence of the factors used in each model. (b) Dark green indicates the actual factor used for model construction and prediction and its time coverage interval. (c) The bright purple curves are the prediction of the model including the GDP factor (based on Pop_M model), which deviated significantly from possible trajectory. QTP: Qinghai-Tibetan Plateau (I), XJ: Xinjiang (II), IMP: Inner-Mongolia Plateau (III), NEPM: Northeast Plain and Mountains (IV), EP: Eastern plain (V), YGP: Yunnan-Guizhou Plateau (VI). Source data are provided as a Source Data file.

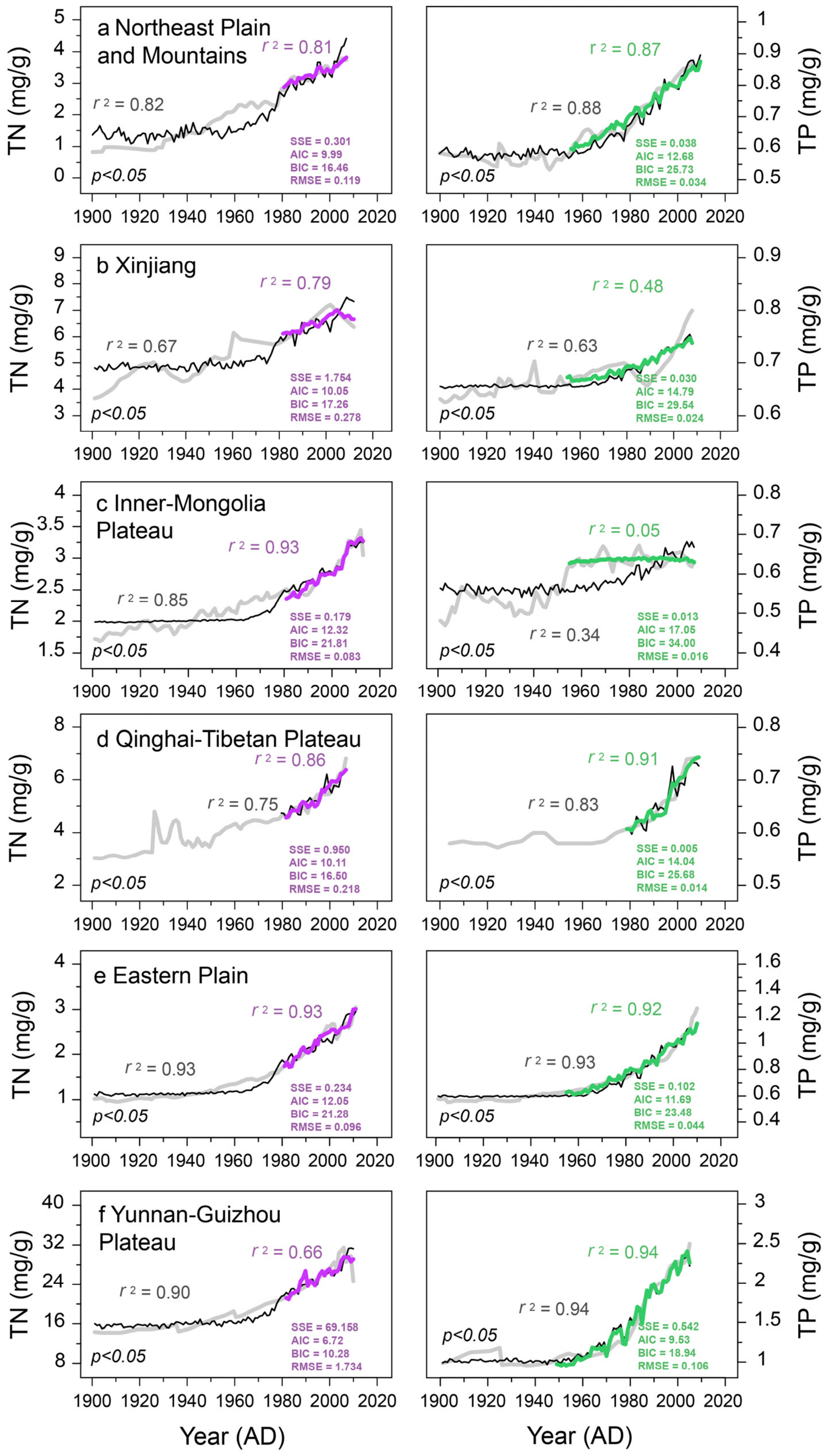


**Supplementary Fig. 13 | Model prediction results of historical changes in sedimentary N, P concentrations in each of the six districts of China (a-f).** The grey curves are the historical mean values of sedimentary N, P concentrations. Black curves are based on climate and fertilizer usage factors. The bold purple and green curves are the model result included the population and N deposition factors, which are used for prediction. All models are significant at the 0.05 level. Source Data are provided as a Source Data file.

**Supplementary Tab. 1 | Details of the 69 lake records from China used in this study.**

| ID | Name | Core ID | Long. (°) | Lat. (°) | Altitude | Depth (m) | Reference | DOI |
|---|---|---|---|---|---|---|---|---|
| 1 | Chahei lake | CH13 | 103.87 | 23.67 | 1483 | 0.4 | Klamt, A. 2020[9] | 10.1016/j.ecolind.2019.105662 |
| 2 | Yilong lake | YL08 | 102.57 | 23.69 | 1414 | 4 | Zhuo, Y. 2020[10] | 10.1016/j.scitotenv.2020.140191 |
| 3 | Qilu lake | QL11 | 102.77 | 24.18 | 1796 | 4 | Zhuo, Y. 2020[10] | 10.1016/j.scitotenv.2020.140191 |
| 4 | Xingyun lake | XY13 | 102.78 | 24.34 | 1698 | 5.3 | Liu, Y. Y. 2017[11] | 10.1007/s10933-017-9952-4 |
| 5 | Fuxian lake | FB03 | 102.89 | 24.59 | 1702 | 89.6 | Liu, W. 2014[12] | 10.1016/j.limno.2013.09.003 |
| 6 | Dianchi lake | DC13 | 102.69 | 24.81 | 1886 | 5 | Zhuo, Y. 2020[10] | 10.1016/j.scitotenv.2020.140191 |
| 7 | Yangzonghai lake | YZH15 | 103.00 | 24.90 | 1770 | 22 | Zhuo, Y. 2020[10] | 10.1016/j.scitotenv.2020.140191 |
| 8 | Erhai lake | EH01 | 100.23 | 25.69 | 1965 | 10.6 | Zhuo, Y. 2020[10] | 10.1016/j.scitotenv.2020.140191 |
| 9 | Haixi lake | HXH-GC2-13 | 99.96 | 26.28 | 2130 | 18.5 | Wang, J. Y. 2019[13] | 10.1016/j.watres.2018.10.032 |
| 10 | Hongfeng lake | HF18 | 106.40 | 26.50 | 1238 | 10.5 | Chen, J. G. 2020[14] | 10.1016/j.agee.2020.106862 |
| 11 | Chenghai lake | CH18 | 100.66 | 26.54 | 1501 | 26.5 | Cao, X. F. 2020[15] | 10.1016/j.watres.2020.116077 |
| 12 | Lugu lake | LGS12 | 100.81 | 27.68 | 2685 | 40 | Lin, Q. 2021[16] | 10.1016/j.catena.2021.105240 |
| 13 | Junshan lake | JS15 | 116.32 | 28.55 | 6 | 3 | Wu, J. X. 2019[17] | 10.1134/s1067413619030044 |
| 14 | KemenCo | TPSC2 | 85.95 | 28.69 | 4652 | 4.2 | Lami, A. 2010[18] | 10.1007/s10750-010-0263-2 |
| 15 | Dongting lake | DT13 | 112.96 | 29.33 | 25 | 6.4 | Chen, X. 2016[19] | 10.1002/eco.1637 |
|  | Dongting lake | DT14 | 112.96 | 29.33 | 25 | 6.4 | Chen, X. 2017[20] | 10.1002/hyp.11254 |
| 16 | Huanggai lake | HGH11-02 | 113.56 | 29.73 | 15 | 4.2 | Yao, S.C. 2014[21] | 10.4081/jlimnol.2014.957 |
| 17 | Honghu lake | HN02 | 113.32 | 29.82 | 20 | 1.91 | Gui, Z.F. 2013[22]<br>Xue, B. 2010[23] | 10.1007/s10750-012-1365-9<br>10.1016/j.quaint.2010.02.024 |
| 18 | Wanghu lake | WHA07 | 115.34 | 29.86 | 9 | 3.7 | Shit, X.L. 2010[24] | 10.13249/j.cnki.sgs.2010.05.009 |
| 19 | Wushan lake | WS07 | 115.59 | 29.91 | 6 | 3.1 | Zhang, E.L. 2010[25] | 10.4081/jlimnol.2010.235 |
| 20 | Taibai lake | TB-1-16 | 115.81 | 30.00 | 5 | 3.2 | Zhang, Y D. 2019[26] | 10.1016/j.chemosphere.2019.06.179 |
| 21 | Longgan lake | LL-4-02 | 116.03 | 30.01 | 2 | 3.8 | Wu, Y. H. 2007[27] | 10.1007/s10933-007-9123-0 |
| 22 | Futou lake | FT14 | 114.21 | 30.05 | 20 | 1.65 | Cao, Y. M. 2020[28] | 10.1016/j.scitotenv.2020.139309 |
| 23 | Sanliqi lake | SLQ12 | 114.95 | 30.10 | 20 | 2 | Cao, Y. M. 2016[29] | 10.1007/s10750-016-2810-y |
| 24 | Liangzi lake | LZ11 | 114.62 | 30.17 | 12 | 4.16 | Zhang, Q.F. 2016[30] | 10. 18307 /2016. 0310 |
| 25 | Shahu lake | SH11 | 114.34 | 30.57 | 20 | 2.5 | Cao, Y. M. 2020[28] | 10.1016/j.scitotenv.2020.139309 |
| 26 | Yanxi lake | YX11 | 114.48 | 30.57 | 20 | 1.9 | Cao, Y. M. 2020[28] | 10.1016/j.scitotenv.2020.139309 |
| 27 | NamCo lake | TPCC3 | 90.93 | 30.77 | 4627 | 21.6 | Lami, A. 2010[18] | 10.1007/s10750-010-0263-2 |
| 28 | Taihu lake | ZS | 120.16 | 31.08 | 8 | 1.9 | Gui, Z.F. 2013[22]<br>Xue, B. 2010[23] | 10.1007/s10750-012-1365-9<br>10.1016/j.quaint.2010.02.024 |
|  | Taihu lake | THS | 120.02 | 31.38 | 8 | 1.9 | Gui, Z.F. 2013[22]<br>Xue, B. 2010[23] | 10.1007/s10750-012-1365-9<br>10.1016/j.quaint.2010.02.024 |
|  | Taihu lake | TM04 | 120.20 | 31.50 | 8 | 1.9 | Wu, J. L. 2007[31] | 10.1007/s10933-006-9058-x |
| 29 | Dianshan lake | DS3-10 | 120.96 | 31.12 | 8 | 2.1 | Li, X. P. 2012[32] | 10.13227/j.hjkx.2012.10.019 |
| 30 | Nanyi lake | NY08 | 118.92 | 31.22 | 3 | 2.25 | Gui, Z.F. 2013[22] | 10.1007/s10750-012-1365-9 |
| 31 | CuoE lake | TPCB3 | 91.49 | 31.42 | 4440 | 8.4 | Lami, A. 2010[18] | 10.1007/s10750-010-0263-2 |
| 32 | Shijiu lake | SJ08 | 118.78 | 31.46 | 3 | 4.08 | Gui, Z.F. 2013[22]<br>Xue, B. 2010[23] | 10.1007/s10750-012-1365-9<br>10.1016/j.quaint.2010.02.024 |
| 33 | Chaohu lake | C14-09 | 117.63 | 31.53 | 0 | 3 | Zan, F.Y. 2012[33] | 10.1039/c1em10760g |
|  | Chaohu lake | CH02 | 117.60 | 31.55 | 0 | 2.7 | Gui, Z.F. 2013[22]<br>Xue, B. 2010[23] | 10.1007/s10750-012-1365-9<br>10.1016/j.quaint.2010.02.024 |
| 34 | Changdang lake | CD-1-16 | 119.55 | 31.62 | 5 | 1.2 | Zhang, Y D. 2018[34] | 10.1016/j.scitotenv.2017.09.185 |

| ID | Name | Core ID | Long. (°) | Lat. (°) | Altitude | Depth (m) | Reference | DOI |
|---|---|---|---|---|---|---|---|---|
| 35 | CuoNa lake | TPCA1 | 91.51 | 32.05 | 4595 | 12.4 | Lami, A. 2010[18] | 10.1007/s10750-010-0263-2 |
| 36 | Rhino lake | XNH08 | 103.89 | 33.18 | 2329 | 0.65 | Li, S. G. 2014[35] | 10.1016/j.quaint.2014.07.069 |
| 37 | Guanshantianchi lake | GSTC21 | 106.31 | 35.26 | 2430 | 10 | This study | |
| 38 | Dongping lake | DP08 | 116.20 | 36.02 | 40 | 3 | Chen, Y. Y. 2014[36] | 10.1007/s12665-014-3223-8 |
| 39 | Qinghai lake | TPNA3 | 100.25 | 36.72 | 3191 | 25.3 | Lami, A. 2010[18] | 10.1007/s10750-010-0263-2 |
| | Qinghai lake | QH02 | 100.03 | 36.78 | 3191 | 22 | Wang, Q. G. 2019[37] | 10.1007/s10653-019-00244-3 |
| 40 | Gahai lake | TPNC3 | 97.55 | 37.14 | 2845 | 9 | Lami, A. 2010[18] | 10.1007/s10750-010-0263-2 |
| 41 | Keluke lake | TPNB3 | 96.88 | 37.29 | 2800 | 8.3 | Lami, A. 2010[18] | 10.1007/s10750-010-0263-2 |
| 42 | Gouchi lake | GC20 | 107.50 | 37.74 | 1295 | 2.5 | This study | |
| 43 | Yueliang lake | YL20a | 105.15 | 38.46 | 1295 | 4 | Ji, P. P. 2023[1] | 10.1016/j.jhazmat.2023.130972 |
| 44 | Baiyangdian lake | BYD11 | 115.92 | 38.84 | 12 | 2 | Guo, W. 2015[38] | 10.1016/j.ecolind.2015.04.019 |
| 45 | Gonghai lake | GH20a | 112.24 | 38.91 | 1860 | 10 | Ji, P. P. 2023[1] | 10.1016/j.jhazmat.2023.130972 |
| 46 | Tiane lake | TE21 | 97.92 | 39.24 | 2330 | 14.5 | This study | |
| 47 | Nalin lake | NL21 | 106.65 | 40.53 | 1025 | 4 | This study | |
| 48 | Daihai lake | DH20 | 112.68 | 40.58 | 1210 | 18 | This study | |
| 49 | Chenpu lake | CP2-12 | 106.82 | 40.65 | 1033 | 3.2 | Wang, J. Z. 2015[39] | 10.1007/s12665-014-3857-6 |
| 50 | Wuliangsu lake | WLS15 | 108.92 | 40.93 | 1014 | 0.86 | He, Y. X. 2015[40] | 10.1177/0959683615585841 |
| 51 | Bosten lake | BST16 | 86.83 | 41.92 | 1050 | 14 | Liu, W. 2019[41] | 10.4081/jlimnol.2019.1875 |
| 52 | Dalinaoer lake | DL-1-15 | 116.66 | 43.32 | 1226 | 8 | Zhen, Z.L. 2019[42] | 10.1134/s0097807819020180 |
| 53 | Chaiwopu lake | CW2-08 | 87.90 | 43.49 | 1904 | 2 | Ma, L. 2013[43] | 10.1007/S00531-012-0838-4 |
| 54 | Sayram lake | S02-10 | 81.13 | 44.65 | 2069 | 92 | Liu, W. 2014[44] | 10.1080/11035897.2014.918170 |
| 55 | Ailike lake | ALK13 | 85.82 | 45.96 | 278 | 4.5 | Lan, B. 2018[45] | 10.1016/j.catena.2017.10.020 |
| 56 | Dalonghupao lake | DLHP15 | 124.38 | 46.45 | 127 | 3.5 | Bao, K. S. 2021[46] | 10.1016/j.envpol.2020.116345 |
| 57 | Xiaolonghupao lake | XLHP15 | 124.28 | 46.47 | 127 | 3.8 | Bao, K. S. 2021[46] | 10.1016/j.envpol.2020.116345 |
| 58 | Nanshan lake | NSH15 | 123.53 | 46.50 | 127 | 2.5 | Bao, K. S. 2021[46] | 10.1016/j.envpol.2020.116345 |
| 59 | Amuta lake | AMT15 | 124.05 | 46.57 | 127 | 4.2 | Bao, K. S. 2021[46] | 10.1016/j.envpol.2020.116345 |
| 60 | Huoshaoheipao lake | HSP15 | 124.16 | 46.59 | 127 | 4.5 | Bao, K. S. 2021[46] | 10.1016/j.envpol.2020.116345 |
| 61 | Xihulu lake | XHL15 | 124.15 | 46.71 | 127 | 3.5 | Bao, K. S. 2021[46] | 10.1016/j.envpol.2020.116345 |
| 62 | Talahong lake | TLH15 | 124.14 | 46.77 | 127 | 3.2 | Bao, K. S. 2021[46] | 10.1016/j.envpol.2020.116345 |
| 63 | Delong lake | DL10 | 124.07 | 46.82 | 127 | 4 | Guan, Y. 2014[47] | 10.1007/s10646-014-1198-6 |
| 64 | Longjiang lake | LJP15 | 123.15 | 46.86 | 127 | 2.2 | Bao, K. S. 2021[46] | 10.1016/j.envpol.2020.116345 |
| 65 | Keqin lake | KQP15 | 124.30 | 47.31 | 127 | 2.5 | Bao, K. S. 2021[46] | 10.1016/j.envpol.2020.116345 |
| 66 | Hongyan lake | HYP15 | 123.39 | 47.35 | 127 | 0.5 | Bao, K. S. 2021[46] | 10.1016/j.envpol.2020.116345 |
| 67 | Wudalianchi-3 lake | wdlc-3 | 126.22 | 48.73 | 266 | 10 | Gui, Z.F. 2012[48] | 10.1016/j.quaint.2012.05.012 |
| 68 | Wudalianchi-5 lake | wdlc-5 | 126.14 | 48.78 | 266 | 6 | Gui, Z.F. 2012[48] | 10.1016/j.quaint.2012.05.012 |
| 69 | Shuanghu lake | SH21 | 87.03 | 48.88 | 1523 | 14.5 | This study | |

**Supplementary Tab. 2 | List of the datasets used in this study.**

| Content | | Source |
|---|---|---|
| **Climate data** | CRU | Temperature and precipitation gridded data for global and regional domains derived from in-situ and satellite observations (copernicus.eu) |
| | CMIP6 climate projection | CMIP6 climate projections (copernicus.eu) |
| **Fertilizer usage data** | Global fertilizer use | ESSD - Global nitrogen and phosphorus fertilizer use for agriculture production in the past half century: shifted hot spots and nutrient imbalance (copernicus.org) |
| | Fertilizer and livestock farming of China | 国家数据 National Data (stats.gov.cn) |
| | Future trend of fertilizer consumption | Emerging human infectious diseases and the links to global food production \| Nature Sustainability |
| **N emissions data** | EDGRA | EDGAR - The Emissions Database for Global Atmospheric Research (europa.eu) |
| | ESGF input4MIPS | input4MIPs Data Search \| input4MIPs \| ESGF-CoG (llnl.gov) |
| | N emission of China | Managing nitrogen to restore water quality in China |
| **Population data** | Population of China | Provincial and gridded population projection for China under shared socioeconomic pathways from 2010 to 2100 |
| **LUCC data** | Global LUCC | Global LULC projection dataset from 2020 to 2100 at a 1km resolution |
| **GDP data** | Global GDP | Gross domestic product (GDP) downscaling: a global gridded dataset consistent with the Shared Socioeconomic Pathways |

**Supplementary Tab. 3 | Correlations between factors involved in model construction and other factors.** The red font indicates that the result is significant at the 0.05 level. Prep is Precipitation, Temp is temperature, Nfert is N fertilizer, Pfert is P fertilizer, Pop is population, Nerur is rural N emission, Neurb is urban N emission, Neagro is N emission from agriculture (cropland), Graz is livestock farming (breeding quantity), Ltem is lake temperature, Crop is cropland percentage in the lake basin, Ndep is N deposition. QTP: Qinghai-Tibetan Plateau (I), XJ: Xinjiang (II), IMP: Inner-Mongolia Plateau (III), NEPM: Northeast Plain and Mountains (IV), EP: Eastern plain (V), YGP: Yunnan-Guizhou Plateau (VI).

| NEPM | N model | | | | | | | | | | | | |
|---|---|---|---|---|---|---|---|---|---|---|---|---|---|
| *r* (pearson) | Pr | Tas | Nfert | Pop | GDP | Nerur | Neurb | Neagro | Graz | Ltem | Crop | Ndep | N |
| Prep | 1 | 0.21064 | -0.09461 | -0.04673 | -0.05329 | -0.13006 | -0.17552 | -0.14567 | 0.00841 | -0.01945 | 0.04159 | -0.17854 | -0.10168 |
| Temp | 0.21064 | 1 | -0.11485 | -0.02029 | 0.25369 | 0.24538 | 0.10912 | 0.29965 | 0.23587 | 0.38426 | 0.36435 | 0.23767 | -0.30106 |
| Nfert | -0.09461 | -0.11485 | 1 | 0.89067 | 0.80793 | 0.94187 | 0.93936 | 0.86763 | 0.86049 | 0.37199 | 0.87647 | 0.89579 | 0.85092 |
| Pop | -0.04673 | -0.02029 | 0.89067 | 1 | 0.57932 | 0.81449 | 0.9271 | 0.78671 | 0.85749 | 0.41756 | 0.86016 | 0.95611 | 0.88493 |
| Ndep | -0.17854 | 0.23767 | 0.89579 | 0.95611 | 0.79286 | 0.94888 | 0.96049 | 0.73976 | 0.93827 | 0.51525 | 0.92154 | 1 | 0.86745 |
| NEPM | P model | | | | | | | | | | | | |
| *r* (pearson) | Pr | Tas | Pfert | Pop | GDP | Graz | Ltem | Crop | P | | | | |
| Prep | 1 | 0.21064 | -0.09792 | -0.09465 | -0.06867 | -0.01446 | 0.09467 | -0.03267 | -0.11709 | | | | |
| Temp | 0.21064 | 1 | -0.09066 | -0.05026 | 0.25784 | 0.29428 | 0.44972 | 0.34981 | -0.24288 | | | | |
| Pfert | -0.09792 | -0.09066 | 1 | 0.87066 | 0.80417 | 0.93426 | 0.35067 | 0.95185 | 0.93515 | | | | |
| Pop | -0.09465 | -0.05026 | 0.87066 | 1 | 0.58073 | 0.85402 | 0.38754 | 0.86016 | 0.87001 | | | | |
| XJ | N model | | | | | | | | | | | | |
| *r* (pearson) | Pr | Tas | Nfert | Pop | GDP | Nerur | Neurb | Neagro | Graz | Ltem | Crop | Ndep | N |
| Prep | 1 | 0.02871 | 0.03901 | 0.11013 | 0.12626 | 0.1381 | 0.1253 | 0.14286 | 0.17531 | 0.29549 | 0.18314 | 0.16877 | -0.06559 |
| Temp | 0.02871 | 1 | 0.29055 | 0.42464 | 0.449 | 0.48271 | 0.4467 | 0.37431 | 0.37526 | 0.28539 | 0.40344 | 0.27214 | 0.17823 |
| Nfert | 0.03901 | 0.29055 | 1 | 0.96815 | 0.80734 | 0.92814 | 0.96741 | 0.87184 | 0.6184 | 0.62957 | 0.88981 | 0.71693 | 0.8158 |
| Pop | 0.11013 | 0.42464 | 0.96815 | 1 | 0.73966 | 0.91806 | 0.95925 | 0.84365 | 0.80963 | 0.64415 | 0.92593 | 0.83936 | 0.8564 |
| Ndep | 0.16877 | 0.27214 | 0.71693 | 0.83936 | 0.55294 | 0.82471 | 0.87901 | 0.59771 | 0.7503 | 0.55235 | 0.87146 | 1 | 0.82229 |
| XJ | P model | | | | | | | | | | | | |
| *r* (pearson) | Pr | Tas | Pfert | Pop | GDP | Graz | Ltem | Crop | P | | | | |
| Prep | 1 | 0.02547 | 0.04934 | 0.09324 | 0.08963 | 0.18797 | 0.30299 | 0.17462 | 0.02464 | | | | |
| Temp | 0.02547 | 1 | 0.24078 | 0.36288 | 0.37844 | 0.41663 | 0.25545 | 0.31282 | 0.23452 | | | | |
| Pfert | 0.04934 | 0.24078 | 1 | 0.96625 | 0.83581 | 0.8858 | 0.70406 | 0.8792 | 0.79686 | | | | |
| Pop | 0.09324 | 0.36288 | 0.96625 | 1 | 0.77177 | 0.9141 | 0.64168 | 0.91335 | 0.6948 | | | | |
| IMP | N model | | | | | | | | | | | | |
| *r* (pearson) | Pr | Tas | Nfert | Pop | GDP | Nerur | Neurb | Neagro | Graz | Ltem | Crop | Ndep | N |
| Prep | 1 | -0.01786 | -0.1399 | -0.18281 | 0.00453 | -0.10153 | -0.14156 | -0.17924 | 0.06465 | 0.17007 | -0.15553 | -0.00649 | -0.10949 |
| Temp | -0.01786 | 1 | 0.28646 | 0.38652 | 0.50881 | 0.51884 | 0.49924 | 0.48056 | 0.4279 | 0.35671 | -0.50298 | 0.41088 | 0.26965 |
| Nfert | -0.1399 | 0.28646 | 1 | 0.93411 | 0.81187 | 0.93864 | 0.9735 | 0.96101 | 0.93438 | 0.33652 | -0.74267 | 0.86296 | 0.92882 |
| Pop | -0.18281 | 0.38652 | 0.93411 | 1 | 0.6028 | 0.86566 | 0.91778 | 0.94203 | 0.79446 | 0.45875 | -0.60928 | 0.95912 | 0.83564 |
| Ndep | -0.00649 | 0.41088 | 0.86296 | 0.95912 | 0.69167 | 0.93947 | 0.93922 | 0.86241 | 0.86802 | 0.42953 | -0.6869 | 1 | 0.83348 |
| IMP | P model | | | | | | | | | | | | |
| *r* (pearson) | Pr | Tas | Pfert | Pop | GDP | Graz | Ltem | Crop | P | | | | |
| Prep | 1 | -0.02036 | -0.13514 | -0.19163 | -0.01866 | 0.04077 | 0.17007 | -0.14127 | -0.10318 | | | | |
| Temp | -0.02036 | 1 | 0.29809 | 0.37732 | 0.51204 | 0.41876 | 0.35671 | -0.49652 | 0.13087 | | | | |
| Pfert | -0.13514 | 0.29809 | 1 | 0.93067 | 0.81762 | 0.9217 | 0.42508 | -0.76412 | 0.78551 | | | | |
| Pop | -0.19163 | 0.37732 | 0.93067 | 1 | 0.60762 | 0.79682 | 0.45875 | -0.60322 | 0.82961 | | | | |

| QTP | N model | | | | | | | | | | | | |
|---|---|---|---|---|---|---|---|---|---|---|---|---|---|
| *r* (pearson) | Pr | Tas | Nfert | Pop | GDP | Nerur | Neurb | Neagro | Graz | Ltem | Crop | Ndep | N |
| Prep | 1 | 0.02625 | 0.37798 | 0.43523 | 0.1519 | 0.24245 | 0.20323 | -0.07405 | -0.13219 | 0.26844 | 0.42429 | 0.38603 | 0.10426 |
| Temp | 0.02625 | 1 | 0.38436 | 0.43882 | 0.48619 | 0.46428 | 0.48133 | 0.22871 | 0.18966 | 0.30232 | 0.44592 | 0.38257 | 0.33435 |
| Nfert | 0.37798 | 0.38436 | 1 | 0.92767 | 0.8272 | 0.91163 | 0.93564 | -0.08734 | 0.03791 | 0.595 | 0.89497 | 0.86601 | 0.87053 |
| Pop | 0.43523 | 0.92767 | 0.92767 | 1 | 0.82799 | 0.91139 | 0.93504 | -0.22644 | 0.03191 | 0.58318 | 0.9757 | 0.82356 | 0.92342 |
| Ndep | 0.38603 | 0.38257 | 0.86601 | 0.82356 | 0.69603 | 0.95288 | 0.9581 | 0.1326 | -0.1614 | 0.5292 | 0.78721 | 1 | 0.86382 |
| QTP | P model | | | | | | | | | | | | |
| *r* (pearson) | Pr | Tas | Pfert | Pop | GDP | Graz | Ltem | Crop | P | | | | |
| Prep | 1 | 0.00668 | 0.30378 | 0.09694 | 0.01356 | -0.17841 | 0.21753 | 0.38086 | 0.05067 | | | | |
| Temp | 0.00668 | 1 | 0.19356 | 0.37229 | 0.26372 | 0.17911 | 0.28586 | 0.34055 | 0.37307 | | | | |
| Pfert | 0.30378 | 0.19356 | 1 | 0.901 | 0.76376 | -0.04346 | 0.56777 | 0.87416 | 0.91303 | | | | |
| Pop | 0.09694 | 0.37229 | 0.901 | 1 | 0.65261 | -0.03726 | 0.54375 | 0.97764 | 0.92278 | | | | |
| EP | N model | | | | | | | | | | | | |
| *r* (pearson) | Pr | Tas | Nfert | Pop | GDP | Nerur | Neurb | Neagro | Graz | Ltem | Crop | Ndep | N |
| Prep | 1 | 0.18146 | -0.27286 | -0.18136 | -0.07267 | -0.22801 | -0.26088 | -0.33311 | -0.31606 | -0.1702 | 0.01117 | -0.13119 | -0.31185 |
| Temp | 0.18146 | 1 | -0.11279 | 0.28565 | 0.41537 | 0.33977 | 0.27809 | 0.12999 | 0.03748 | 0.33737 | -0.53678 | 0.50773 | -0.12513 |
| Nfert | -0.27286 | -0.11279 | 1 | 0.96264 | 0.79229 | 0.94459 | 0.96644 | 0.87679 | 0.17192 | 0.45541 | -0.95067 | 0.96527 | 0.95874 |
| Pop | -0.18136 | 0.28565 | 0.96264 | 1 | 0.82134 | 0.91377 | 0.92592 | 0.82179 | 0.1009 | 0.34305 | -0.96749 | 0.87056 | 0.89744 |
| Ndep | -0.13119 | 0.50773 | 0.96527 | 0.87056 | 0.80053 | 0.96042 | 0.95911 | 0.43143 | 0.23985 | 0.48671 | -0.93332 | 1 | 0.83857 |
| EP | P model | | | | | | | | | | | | |
| *r* (pearson) | Pr | Tas | Pfert | Pop | GDP | Graz | Ltem | Crop | P | | | | |
| Prep | 1 | 0.16221 | -0.2736 | -0.18136 | -0.07267 | -0.31606 | -0.1702 | 0.01117 | -0.29058 | | | | |
| Temp | 0.16221 | 1 | -0.10599 | 0.28565 | 0.41537 | 0.03748 | 0.33737 | -0.53678 | -0.08296 | | | | |
| Pfert | -0.2736 | -0.10599 | 1 | 0.94754 | 0.8017 | 0.06853 | 0.55226 | -0.9207 | 0.96467 | | | | |
| Pop | -0.18136 | 0.28565 | 0.94754 | 1 | 0.8214 | -0.01765 | 0.3443 | -0.96282 | 0.92081 | | | | |
| YGP | N model | | | | | | | | | | | | |
| *r* (pearson) | Pr | Tas | Nfert | Pop | GDP | Nerur | Neurb | Neagro | Graz | Ltem | Crop | Ndep | N |
| Prep | 1 | 0.17429 | -0.42462 | -0.24268 | -0.18449 | -0.17009 | -0.1874 | -0.22494 | -0.12404 | -0.0894 | 0.06821 | 0.06652 | -0.46684 |
| Temp | 0.17429 | 1 | -0.10115 | -0.11652 | 0.08773 | 0.13839 | 0.11481 | 0.05276 | 0.20578 | 0.27747 | -0.16488 | 0.19358 | -0.1012 |
| Nfert | -0.42462 | -0.10115 | 1 | 0.90387 | 0.8239 | 0.92406 | 0.95494 | 0.91798 | 0.80316 | 0.32363 | -0.92724 | 0.94933 | 0.94741 |
| Pop | -0.24268 | -0.11652 | 0.90387 | 1 | 0.67201 | 0.79352 | 0.82458 | 0.85019 | 0.67047 | 0.30433 | -0.76221 | 0.7666 | 0.88003 |
| Ndep | 0.06652 | 0.19358 | 0.94933 | 0.7666 | 0.88889 | 0.93019 | 0.93492 | 0.54424 | 0.81783 | 0.33926 | -0.91398 | 1 | 0.83715 |
| YGP | P model | | | | | | | | | | | | |
| *r* (pearson) | Pr | Tas | Pfert | Pop | GDP | Graz | Ltem | Crop | P | | | | |
| Prep | 1 | 0.19815 | -0.39721 | -0.18359 | -0.06687 | -0.0311 | -0.06574 | -0.11528 | -0.40009 | | | | |
| Temp | 0.19815 | 1 | -0.13551 | -0.17468 | 0.04598 | 0.1463 | 0.34243 | -0.11205 | -0.13811 | | | | |
| Pfert | -0.39721 | -0.13551 | 1 | 0.8934 | 0.81926 | 0.94901 | 0.30322 | -0.88852 | 0.98549 | | | | |
| Pop | -0.18359 | -0.17468 | 0.8934 | 1 | 0.64982 | 0.62685 | 0.20541 | -0.61467 | 0.90283 | | | | |

**Supplementary Tab. 4 | Model fitting results for the six lake districts of China.**

| District | Indicator | Independent variable | Time range | Formula | $r^2$ | $p$ |
|---|---|---|---|---|---|---|
| XJ | TP | Prep, Temp, Pfert, Pop | 1955-2008 | $y_P = -0.000102285x_{prep} + 0.00427x_{temp} + 0.01586x_{pfert} + 0.000232219x_{Pop} + 0.65183$ | 0.48 | <0.05 |
| | TN | Prep, Temp, Nfert, Ndep, Pop | 1981-2011 | $y_N = 0.00133x_{prep} - 0.00355x_{temp} - 0.0048x_{nfert} + 0.24967x_{Pop} + 0.11321x_{ndep} + 4.0568$ | 0.79 | <0.05 |
| IMP | TP | Prep, Temp, Pfert, Pop | 1955-2007 | $y_P = 0.00014604x_{prep} - 0.0015x_{temp} + 0.05911x_{pfert} + 0.0018x_{Pop} + 0.62659$ | 0.05 | <0.05 |
| | TN | Prep, Temp, Nfert, Ndep, Pop | 1981-2012 | $y_N = 0.00371x_{prep} - 0.00858x_{temp} + 0.06308x_{nfert} + 0.04842x_{Pop} - 0.0389x_{ndep} + 1.11162$ | 0.93 | <0.05 |
| EP | TP | Prep, Temp, Pfert, Pop | 1955-2010 | $y_P = -0.00013429x_{prep} + 0.0367x_{temp} + 0.08199x_{pfert} + 0.00029964x_{Pop} + 0.00384$ | 0.92 | <0.05 |
| | TN | Prep, Temp, Nfert, Ndep, Pop | 1981-2011 | $y_N = -0.000097x_{prep} + 0.152390x_{temp} - 0.04287x_{nfert} + 0.001710x_{Pop} + 0.114920x_{ndep} - 2.349430$ | 0.93 | <0.05 |
| YGP | TP | Prep, Temp, Pfert, Pop | 1955-2005 | $y_P = 0.00059183x_{prep} + 0.01884000x_{temp} + 0.35853x_{pfert} - 0.0009804x_{Pop} + 0.74843$ | 0.94 | <0.05 |
| | TN | Prep, Temp, Nfert, Ndep, Pop | 1981-2009 | $y_N = -0.04876x_{prep} + 0.89425x_{temp} - 0.22739x_{nfert} + 0.00345x_{Pop} + 0.89377x_{ndep} + 2.32355$ | 0.66 | <0.05 |
| QTP | TP | Prep, Temp, Pfert | 1979-2009 | $y_P = -0.000190497x_{prep} + 0.0047x_{temp} + 0.00972x_{pfert} + 0.10347x_{Pop} + 0.47419$ | 0.91 | <0.05 |
| | TN | Prep, Temp, Nfert, Ndep | 1981-2006 | $y_N = -0.01423x_{prep} + 0.02286x_{temp} + 0.04711x_{nfert} + 1.35036x_{Pop} + 0.0863x_{ndep} + 2.78071$ | 0.86 | <0.05 |
| NEPM | TP | Prep, Temp, Pfert, Pop | 1955-2010 | $y_P = 0.000453211x_{prep} - 0.00518x_{temp} + 0.04558x_{pfert} + 0.00871x_{Pop} + 0.52843$ | 0.87 | <0.05 |
| | TN | Prep, Temp, Nfert, Ndep, Pop | 1981-2007 | $y_N = 0.000265439x_{prep} - 0.04898x_{temp} + 0.04131x_{nfert} + 0.04038x_{Pop} + 0.02753x_{ndep} + 1.81569$ | 0.81 | <0.05 |

**Note**: XJ: Xinjiang, IMP: Inner Mongolia Plateau, EP: Eastern Plain, YGP: Yunnan-Guizhou Plateau, QTP: Qinghai-Tibetan Plateau, NEPM: Northeast Plain and Mountains. The model for each time series was constructed according to the temporal coverage of each dataset. Prep: precipitation, Temp: temperature, Pfert: P fertilizer consumption, Nfert: N fertilizer consumption, Pop: population, Ndep: N deposition.

## Supplementary References


1. Ji P, et al. Anthropogenic atmospheric deposition caused the nutrient and toxic metal enrichment of the enclosed lakes in North China. *J. Hazard. Mater.* **448**, 130972 (2023).
2. Appleby P, Oldfield F, Thompson R, Huttunen P, Tolonen K. 210 Pb dating of annually laminated lake sediments from Finland. *Nature* **280**, 53–55 (1979).
3. Hu H. The distribution of population in China, with statistics and maps. *Acta Geogr. Sin* **2**, 33–74 (1935).
4. Yu G, et al. Stabilization of atmospheric nitrogen deposition in China over the past decade. *Nat. Geosci.* **12**, 424–429 (2019).
5. Lu C, Tian H. Global nitrogen and phosphorus fertilizer use for agriculture production in the past half century: shifted hot spots and nutrient imbalance. *Earth. Syst. Sci. Data* **9**, 181–192 (2017).
6. Rohr JR, et al. Emerging human infectious diseases and the links to global food production. *Nat. Sustain.* **2**, 445–456 (2019).
7. Crippa M, et al. High resolution temporal profiles in the Emissions Database for Global Atmospheric Research. *Sci. Data* **7**, 121 (2020).
8. Gidden MJ, et al. Global emissions pathways under different socioeconomic scenarios for use in CMIP6: a dataset of harmonized emissions trajectories through the end of the century. *Geosci. Model Dev.* **12**, 1443–1475 (2019).
9. Klamt A-M, Hu K, Huang L, Chen X, Liu X, Chen G. An extreme drought event homogenises the diatom composition of two shallow lakes in southwest China. *Ecol. Indic.* **108**, 105662 (2020).
10. Zhuo Y, Zeng W. Using stable nitrogen isotopes to reproduce the process of the impact of human activities on the lakes in the Yunnan Guizhou Plateau in the past 150-200 years. *Sci. Total. Environ.* **741**, 140191 (2020).
11. Liu Y, et al. Biological responses to recent eutrophication and hydrologic changes in Xingyun Lake, southwest China. *J. Paleolimnol.* **57**, 343–360 (2017).
12. Liu W, Wu J, Zeng H, Ma L. Geochemical evidence of human impacts on deep Lake Fuxian, southwest China. *Limnologica* **45**, 1–6 (2014).
13. Wang J, Chen G, Kang W, Hu K, Wang L. Impoundment intensity determines temporal patterns of hydrological fluctuation, carbon cycling and algal succession in a dammed lake of Southwest China. *Water Res.* **148**, 162–175 (2019).
14. Chen J, Yu J, Bai X, Zeng Y, Wang J. Fragility of karst ecosystem and environment: Long-term evidence from lake sediments. *Agr. Ecosyst. Environ.* **294**, 106862 (2020).
15. Cao X, et al. Sedimentary ancient DNA metabarcoding delineates the contrastingly temporal change of lake cyanobacterial communities. *Water Res.* **183**, 116077 (2020).
16. Lin Q, et al. Organic carbon burial in a large, deep alpine lake (southwest China) in response to changes in climate, land use and nutrient supply over the past ~100 years. *Catena* **202**, 105240 (2021).
17. Wu J, et al. Effects of Rapid Enclosure of Aquatic Ecosystems on Genetic Diversity and Genetic Structure of Daphnia similoides sinensis in a Eutrophic Chinese Lake. *Russ. J. Ecol.* **50**, 289–299 (2019).
18. Lami A, et al. Sedimentary evidence for recent increases in production in Tibetan plateau lakes. *Hydrobiologia* **648**, 175–187 (2010).

19. Chen X, McGowan S, Xu L, Zeng L, Yang X. Effects of hydrological regulation and anthropogenic pollutants on Dongting Lake in the Yangtze floodplain. *Ecohydrology* **9**, 315–325 (2016).
20. Chen X, McGowan S, Zeng L, Xu L, Yang X. Changes in carbon and nitrogen cycling in a floodplain lake over recent decades linked to littoral expansion, declining riverine influx, and eutrophication. *Hydrol. Process.* **31**, 3110–3121 (2017).
21. Yao S, Xue B. Sedimentary geochemical record of human-induced environmental changes in Huanggaihu Lake in the middle reach of the Yangtze River, China. *J. Limnol.* **73**, 31–39 (2014).
22. Gui Z, Xue B, Yao S, Wei W, Yi S. Organic carbon burial in lake sediments in the middle and lower reaches of the Yangtze River Basin, China. *Hydrobiologia* **710**, 143–156 (2013).
23. Xue B, Yao S, Xia W, Zhu Y. Some sediment-geochemical evidence for the recent environmental changes of the lakes from the middle and lower Yangtze River basin, China. *Quatern. Int.* **226**, 29–37 (2010).
24. Shi X, Qin B. Nutrients Distribution Character and Their Influential Factors in Core Sediments from Wanghu Lake in Middle Reaches of Changjiang River(in chinese with english abstract). *Sci. Geol. Sin.* **30**, 766–771 (2010).
25. Zhang E, Liu E, Jones R, Langdon P, Yang X, Shen J. A 150-year record of recent changes in human activity and eutrophication of Lake Wushan from the middle reach of the Yangze River, China. *J. Limnol.* **69**, 235–241 (2010).
26. Zhang Y, Yu J, Su Y, Du Y, Liu Z. Long-term changes of water quality in aquaculture-dominated lakes as revealed by sediment geochemical records in Lake Taibai (Eastern China). *Chemosphere* **235**, 297–307 (2019).
27. Wu Y, Lücke A, Wang S. Assessment of nutrient sources and paleoproductivity during the past century in Longgan Lake, middle reaches of the Yangtze River, China. *J. Paleolimnol.* **39**, 451–462 (2007).
28. Cao Y, et al. Regime shifts in shallow lake ecosystems along an urban-rural gradient in central China. *Sci. Total. Environ.* **733**, 139309 (2020).
29. Cao Y, Zhang E, Tang H, Langdon P, Ning D, Zheng W. Combined effects of nutrients and trace metals on chironomid composition and morphology in a heavily polluted lake in central China since the early 20th century. *Hydrobiologia* **779**, 147–159 (2016).
30. Zhang Q, Dong X, Yang X. Environmental evolution of Lake Liangzi and its driving factors over the past 100 years, Hubei Province. *J. Lake Sci.* **28**, 545–553 (2016).
31. Wu J, Huang C, Zeng H, Schleser GH, Battarbee R. Sedimentary evidence for recent eutrophication in the northern basin of Lake Taihu, China: human impacts on a large shallow lake. *J. Paleolimnol.* **38**, 13–23 (2007).
32. Li X, Chen X, Dong X, Dong Z, Sun D. Nutrient dynamics over the past 100 years and its restoration baseline in Dianshan Lake. *Huan. Jing. Ke. Xue.* **33**, 3301–3307 (2012).
33. Zan F, et al. A 100-year sedimentary record of natural and anthropogenic impacts on a shallow eutrophic lake, Lake Chaohu, China. *J. Environ. Monitor.* **14**, 804–816 (2012).
34. Zhang Y, et al. Sedimentary lipid biomarker record of human-induced environmental change during the past century in Lake Changdang, Lake Taihu basin, Eastern China. *Sci. Total. Environ.* **613-614**, 907–918 (2018).
35. Li S, Hu X, Tang Y, Huang C, Xiao W. Changes in lacustrine environment due

to anthropogenic activities over 240 years in Jiuzhaigou National Nature Reserve, southwest China. *Quatern. Int.* **349**, 367–375 (2014).

36. Chen Y, Chen S, Yu S, Zhang Z, Yang L, Yao M. Distribution and speciation of phosphorus in sediments of Dongping Lake, North China. *Environ. Earth Sci.* **72**, 3173–3182 (2014).
37. Wang Q, Sha Z, Wang J, Du J, Hu J, Ma Y. Historical changes in the major and trace elements in the sedimentary records of Lake Qinghai, Qinghai-Tibet Plateau: implications for anthropogenic activities. *Environ. Geochem. Health* **41**, 2093–2111 (2019).
38. Guo W, Huo S, Ding W. Historical record of human impact in a lake of northern China: Magnetic susceptibility, nutrients, heavy metals and OCPs. *Ecol. Indic.* **57**, 74–81 (2015).
39. Wang J, Wu J, Zeng H. Sediment record of abrupt environmental changes in Lake Chenpu, upper reaches of Yellow River Basin, north China. *Environ. Earth Sci.* **73**, 6355–6363 (2015).
40. He Y, Sun D, Wu J, Sun Y. Factors controlling the past ~150-year ecological dynamics of Lake Wuliangsu in the upper reaches of the Yellow River, China. *Holocene* **25**, 1394–1401 (2015).
41. Liu W, Abuduwaili J, Ma L. Geochemistry of major and trace elements and their environmental significances in core sediments from Bosten Lake, arid northwestern China. *J. Limnol.* **78**, 201–209 (2019).
42. Zhen Z, Zhang S, Li W. Environment Variation in North Margin of East Asian Summer Monsoon Record in Dali Lake over Past 168 Years. *Water Res.* **46**, 172–181 (2019).
43. Ma L, Wu J, Abuduwaili J. Climate and environmental changes over the past 150 years inferred from the sediments of Chaiwopu Lake, central Tianshan Mountains, northwest China. *Int. J. Earth Sci.* **102**, 959–967 (2013).
44. Liu W, Wu J, Ma L, Zeng H. A 200-year sediment record of environmental change from Lake Sayram, Tianshan Mountains in China. *Gff* **136**, 548–555 (2014).
45. Lan B, Zhang D, Yang Y. Lacustrine sediment chronology defined by 137 Cs, 210 Pb and 14 C and the hydrological evolution of Lake Ailike during 1901–2013, northern Xinjiang, China. *Catena* **161**, 104–112 (2018).
46. Bao K, Zhang Y, Zaccone C, Meadows ME. Human impact on C/N/P accumulation in lake sediments from northeast China during the last 150 years. *Environ. Pollut.* **271**, 116345 (2021).
47. Guan Y, Zang S, Xiao H. The vertical variation of nutrients in a sediment core of Delong Lake reveals the anthropogenic effect. *Ecotoxicology* **23**, 480–485 (2014).
48. Gui Z, Xue B, Yao S, Zhang F, Yi S. Catchment erosion and trophic status changes over the past century as recorded in sediments from Wudalianchi Lake, the northernmost volcanic lake in China. *Quatern. Int.* **282**, 163–170 (2012).
